# Spotlight on the diagnosis of extrinsic allergic alveolitis (hypersensitivity pneumonitis)

**DOI:** 10.1186/s12995-015-0057-6

**Published:** 2015-04-20

**Authors:** Xaver Baur, Axel Fischer, Lygia T Budnik

**Affiliations:** Institute for Occupational Medicine, Charité University Medicine Berlin, Berlin, Germany; Division Occupational Toxicology and Immunology, Institute for Occupational and Maritime Medicine (ZfAM), University Medical Center Hamburg-Eppendorf. University of Hamburg, Hamburg, Germany; European Society for Environmental and Occupational Medicine, EOM, Berlin, Germany

## Abstract

Repeated inhalative exposures to antigenic material from a variety of sources, mainly from moulds, thermophilic *Actinomycetes*, and avians, respectively, can induce immune responses with the clinical picture of extrinsic allergic alveolitis (EAA) or hypersensitivity pneumonitis. Delays of years or even decades till the diagnosis is made are not uncommon; frequent misdiagnoses include allergic asthma, COPD, recurrent flue and other infections. We provide here the state of the art references, a detailed case description and recommend a current diagnostics schema.

Extrinsic allergic alveolitis (EAA) or hypersensitivity pneumonitis is an immunologically mediated disease caused by sensitization to repeated inhalation of antigenic organic material derived from a variety of sources, such as mouldy hay, mouldy wood bark, bacterially contaminated metal working fluid or humidifier water (Figures 1, 2, 3 and 4), avian bloom proteins, but also chemicals such as isocyanates may cause this disorder [1-8], (Table 1). EAA is predominantly an occupational disease with most common antigens from thermophilic *Actinomycetes* species, various moulds and avian proteins. The prevalence of extrinsic allergic alveolitis varies depending on the climate, geographic conditions, occupational and industry factors, and is mostly is in the range of 0.1-3 [9-11] percent with farmer's lung or bird fancier’s lung as the prevailing one in most regions. Sensitized subjects may develop an acute, subacute (intermittent), and chronic progressive course depending on degree of sensitisation, intensity and duration of exposure.

**Figure 1:**
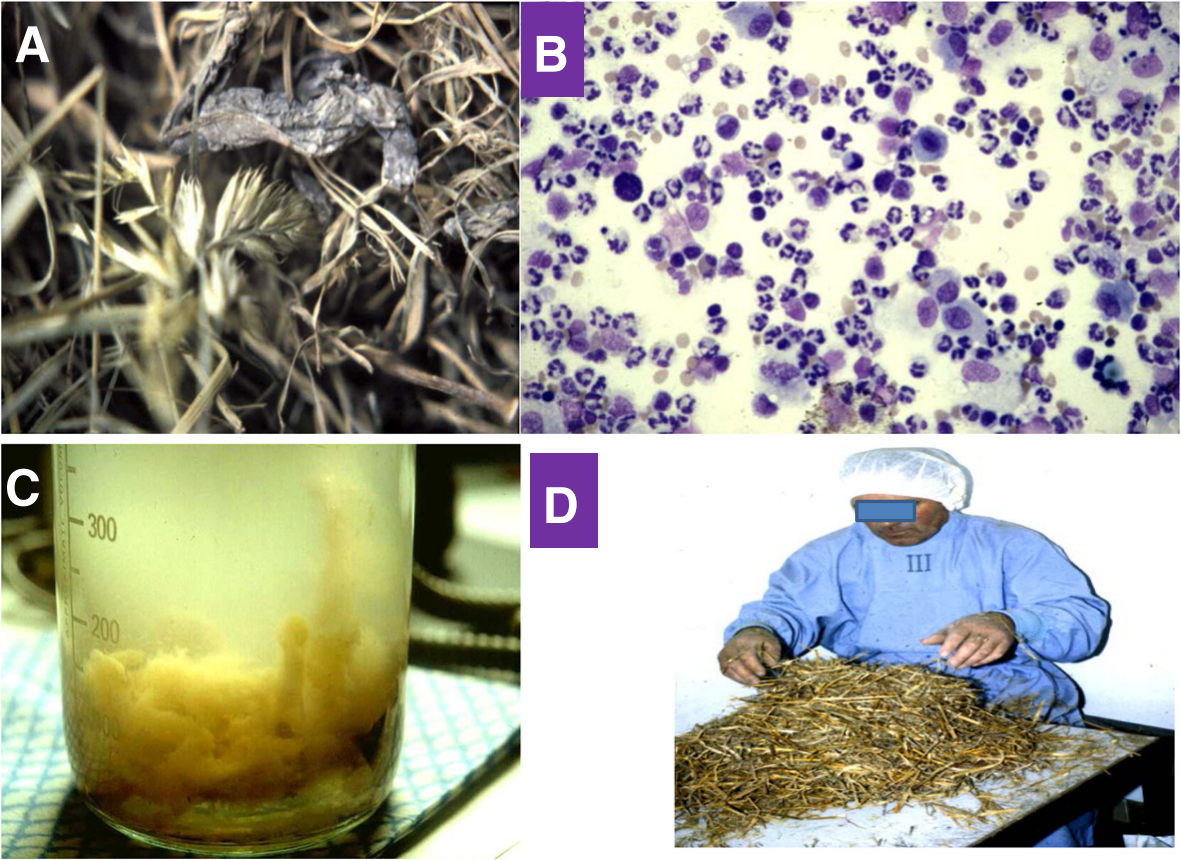
Agents causing extrinsic allergic alveolitis, specific inhalative challenge testing, and bronchoalveolar lavage cells. **A**. Mouldy hay causing farmer’s lung disease in the subject shown in Figure 1D and Figure 4 **B**. Bronchoalveolar lavage with extensive neutrophilia present six hours post challenge in an subject with pigeon breeder’s lung. **C**. Water and sediment of a humidifier water of a printing plant (of the subject shown in Figure 3). Microscopic examinations demonstrated a variety of bacteria and moulds in this specimen. **D**. Occupational type specific inhalative challenge test with mouldy hay (Figure 1A).

**Figure 2:**
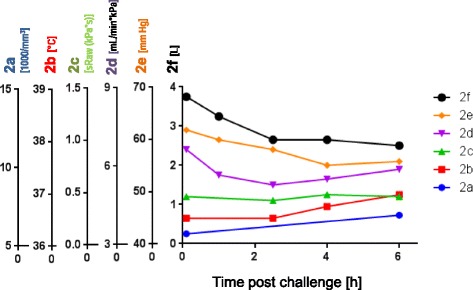
Acute extrinsic allergic alveolitis after indicative challenge by mouldy hay. The figure shows a summary of the clinical diagnostic findings of the patient described as case 1. Clinical evidence: Several hours post challenge, fever, malaise, cough, associated with a restrictive ventilation pattern, and impaired gas exchange. The data shows lung function and other clinical findings in a time scale after the challenge [h]: **f**: Vital capacity VC [L], **e**: P_a,O2_ arterial oxygen pressure [mmHg], **d**. Diffusion capacity/transfer coefficient for carbon monoxide (T_L,CO_) factor [mL/min*kPa], **c**: Specific airway resistance (sRaw) measured by whole body plethysmography [kPa*s]; Further parameter shown are: **b**: Temperature profile gradation [°C], **a**. Blood leukocytes counts *1000/mm^3^].

**Figure 3:**
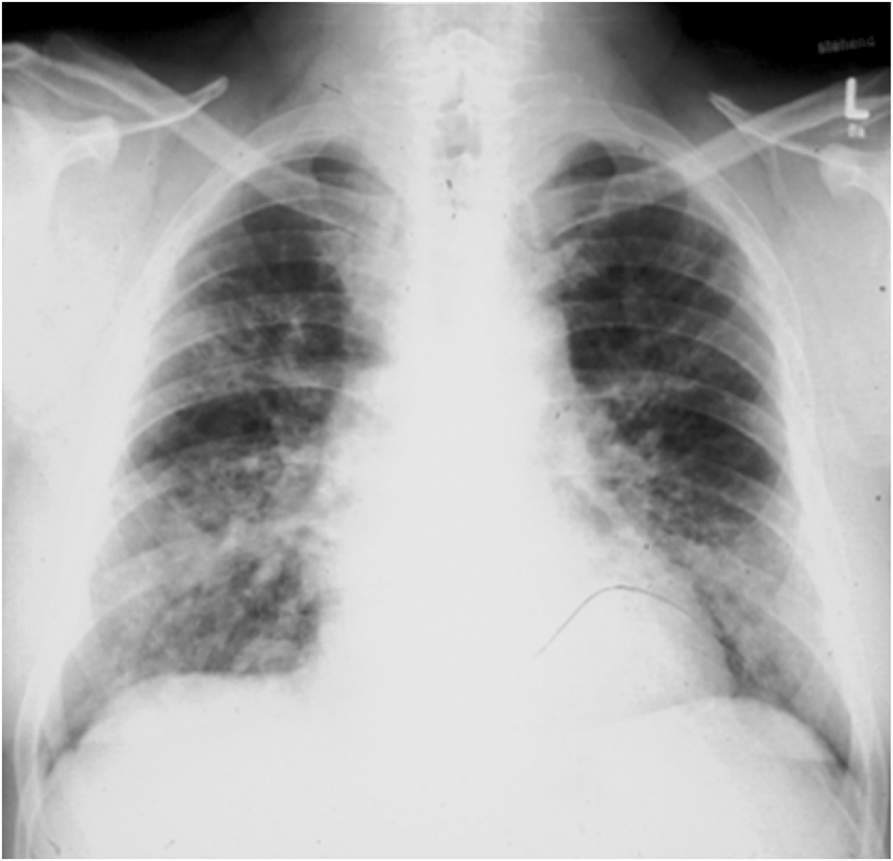
Chest x-ray of a 33 year old worker of a printing plant, suffering from subacute humidifier lung disease. For details see text. There are patchy infiltrates predominantly in the lower and middle lung fields.

**Figure 4:**
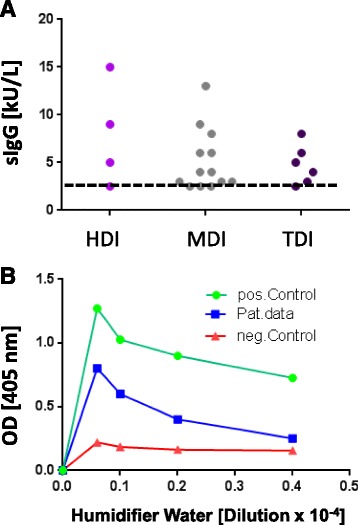
Presence of specific IgG antibodies in blood sera of 23 subjects suffering from isocyanate alveolitis. Note that all but five subjects show significant levels of such antibodies. The diisocyanates toluylene disocyanate (TDI), methylene diphenyl diisocyanate (MDI) and hexamethylene diisocyanate (HDI) bound to human serum albumin (HSA) were used for immune absorbent assay (CAP immunoanalysis) as described. For methodological details see Baur [4], Budnik et al. [18]. Specific IgG antibodies in blood sera from a patient suffering from humidifier lung due to exposure to contaminated humidifier water (Figure 1C). Shown is ELISA assay with anti IgG antibodies recognising dose specific reaction to various concentrations of workplace related humidifier water antigens. The negative and positive controls show lab intern positive and negative samples from patients with strong signal (positive reaction) and pool serum from healthy subjects without contact with humidifier aerosols (negative control, unspecific binding).

**Table 1:** **Sources and major antigens of extrinsic allergic alveolitis (hypersensitivity pneumonitis)**

**Disease**	**Causative exposure**	**Antigens**
Farmer's lung	Mouldy hay	*Saccharopolyspora rectivirgula* (*Micropolyspora faeni*), *Thermoactinomyces vulgaris,* Aspergilli
Bagassosis	Mouldy sugar cane fiber	*Thermoactinomyces sacchari*
Humidifier/air-conditioner lung	Contaminated forced-air systems, heated water reservoirs	*S. rectivirgula, T. vulgaris,* various fungi
Bird breeder's lung	Pigeons, parakeets, fowl	Avian proteins (of bloom or faeces)
Metal working hypersensitivity pneumonitis	Microbially contaminated metal working fluid	Various moulds and bacteria
Cheese worker's lung	Cheese mould	*Penicillium casei*
Malt worker‘s lung	Mouldy malt	*Aspergillus clavatus*
Paprika splitter‘s lung	Paprika dust	*Mucor stolonifer*
Mollusk shell hypersensitivity	Shell dust	Proteins in dust from sea snail shells or mother-of-pearl shells
Chemical worker’s lung, isocyanate alveolitis	Manufacture of plastics, polyurethane foam, rubber	Trimellitic anhydride, diisocyanates

EAA is associated with diffuse inflammation of lung parenchyma and airways in sensitized subjects including non-caseating interstitial granulomas and peribronchial mononuclear cell infiltration with giant cells. Although most affected subjects typically have high serum concentrations of circulating immunoglobulin G antibodies specific for the causative antigen(s), the diagnosis may be difficult as the symptoms are often non-specific, have typically a latency period of several hours, or may appear constitutional with malaise and weight loss; frequently they are gradually progressive over years, finally with persisting respiratory distress independent of the inducing exposure (Figures 1, 2, 3 and 4). Delays of years or even decades till the diagnosis is made are not uncommon, misdiagnoses include allergic asthma, COPD, recurrent flue and other infections. We recommend the following diagnostics schema for the disease pattern.

## Diagnostics (Table 2) [4,12]

**Table 2:** **Diagnostic parameters of extrinsic allergic alveolitis**

**Symptoms:**	
•	Exposure (work)-related cough, chest tightness, dyspnea, fever, with latency period of several hrs
•	Progressive flu-like symptoms during the exposure periods (e.g. working week) with solution at days off
•	Dyspnea on exertion
•	Weight loss in the absence of any other reason
**Clinical/physical examination:**	
•	Fine bibasilar end-inspiratory crackles in advanced chronic forms clubbing and respiratory distress
**Serology:**	
•	Presence of high serum concentrations of antigen-specific IgG antibodies
**Lung function testing:**	
•	FVC < 80% predicted (below lower limit of normal) or
•	FVC < 70% pred. and/or T_L,CO_ < 80% pred. or
•	T_L,CO_ < 60% pred. or hypoxemia during exercise
**Radiology:**	
•	Abnormal chest x-ray (nodular, patchy and/or diffuse ground glass pattern)
•	Abnormal HRCT (ground glass, nodular and/ or patchy opacities, mosaic or UIP pattern
**Serial lung function testing and clinical investigations during antigen exposure periods and days off** (for minimal diagnostic changes see SIC below):	
**Specific inhalation challenge (minimal changes after 5-12 hrs):**	
•	FVC and TLC -15%
•	T_L,CO_ -15% or P_a,O2_ – 7 mmHg
•	New fine bibasilar end-inspiratory crackles
•	Systemic symptoms (temperature + 1°C and leukocytosis + 2.5 × 10^9^/L)

1. **Case history:** 4-8 hours following heavy exposure to an inciting agent patients develop fever, chills, malaise, cough, dyspnea, headache (acute course). Some cases don’t develop acute symptoms, rather, they have an insidious onset of these symptoms. Especially at lower exposures, patients gradually develop productive chronic cough, dyspnea on exertion, fatigue, anorexia, weight loss (subacute or intermittent course) (Table 2). These findings may be also present in patients who experience repeated acute attacks.
2. **Physical examination:** During the acute attack there are fever, tachypnea, and diffuse fine bibasilar end-inspiratory crackles upon auscultation. Further frequent findings include muscle wasting, weight loss, in the chronic pronounced form also clubbing, tachypnea, respiratory distress.
3. **Lung function testing:** A restrictive ventilatory pattern (i.e. reduced total lung capacity, vital capacity, and lung compliance) and impaired gas exchange parameters (reduced diffusing capacity, hypoxemia during exercise or even at rest) are typical features of the advanced chronic course. Also a mixed obstructive and restrictive ventilation pattern can develop.
4. **IgG antibodies and other laboratory findings:** High serum concentrations of IgG antibodies specific for causative antigens are found in c. 70% of affected patients (Figure 2). More problematic from the diagnostic view is that more than 50% of the prevailing exposed healthy subjects may show such antibodies, depending on the causative antigen(s) and sensitivity and specificity of the laboratory test used. So far, for most causative antigens validated laboratory tests are not available on the market. In addition to an immunocomplex-mediated process, cell-mediated immunity obviously plays an important pathophysiological role. However, no routine diagnostic test for displaying this cellular response is available yet. Other abnormal laboratory findings during acute and subacute courses include leukocytosis, neutrophilia, elevated erythrocyte sedimentation rate, and increased levels of quantitative immunoglobulins and C-reactive protein.
5. **Radiological findings:** The typical pictures are centrilobular micronodular (HRCT), patchy or reticular opacities which are mostly prominent in lower lung zones (Figure 3). Ground-glass opacities in the lower and middle lung zones and an interstitial pneumonitis-like pattern may be present during acute attacks. The end stage of the chronic course is characterized by fibrosis and even honeycombing predominant in the lower parts of the lungs. Emphysema may also occur [13].
6. Serial lung function testing during antigen exposure periods and days off (exhibiting changes as outlined under SIC and restitution of symptoms and impaired lung function during days off).
7. **Specific inhalative challenge test (SIC):** This is a laborious and time consuming diagnostic test by the use of the suspected causative agent. It should only be performed by well-experienced physicians. If done correctly it is a very sensitive and specific diagnostic method. See Table 2 and example in Figures. 1D, methodological methods have been published elsewhere [14,15], with the supplementary material from the European task force on SIC published online asthma additional Handbook of procedures for specific inhalation challenge testing http://erj.ersjournals.com/content/suppl/2014/03/07/09031936.00180313.DC1/Final_Handbook.pdf
8. **Bronchoalveolar lavage:** Bronchoalveolar lavage shows leukocytosis (neutrophilia) in the alveoli and small airways in the acute course followed by an influx of mononuclear cells (Figure 1D). In the subacute and chronic courses lymphocytosis with increased CD8 + cells and a CD4+/CD8+ ratio of < 1 are observed 6 hours post challenge.
9. **Lung histology:** Invasive diagnostics, i.e. lung biopsy, is usually not needed. In the acute course leukocytic inflammation is dominating. Lymphocyte-dominant interstitial inflammatory cell infiltration, well-formed interstitial epitheliod cell nonnecrotizing granulomas with giant cells are characteristic for the subacute and chronic courses. Cellular bronchiolitis and foci of bronchiolitis obliterans and intra-alveolar fibrosis may also occur. The chronic course includes an interstitial (organizing) pneumonia–like pattern with sub pleural patchy fibrosis, fibroblastic foci with centrilobular fibrosis, finally alveolar destruction (honeycombing) [16,17].
10. **Integrated diagnostic approach:** Above steps 1 through 5 present basic routine diagnostics. If all of them exhibit concordant positive findings diagnosis of extrinic allergic alveolitis is sufficiently likely. If one or more of the respective 6 parameters is/are negative or equivocal at least one additional diagnostic procedure is needed, i.e. serial lung function testing during antigen exposure periods and days off (combined with follow-up of clinical symptoms) or specific inhalation challenge test, bronchoalveolar lavage or lung biopsy (Table 3).

**Table 3:** **Summary of diagnostic criteria**

➢	Routine, basic diagnostics:
•	Case history: Exposure to relevant antigen(s)
•	Exposure-related respiratory and systemic symptoms
•	Specific IgG antibodies to relevant antigens (i.e. antigen-HSA-conjugates)
•	Bibasilar end-inspiratory crackles (lower lung)
•	Lung nodular, patchy and/or ground glass pattern in chest x-ray or HRCT
•	Restrictive ventilation pattern (FVC, TLC) and reduced gas exchange parameters (T_L,CO_; P_a,O2_)
➢	Facultative diagnostic parameter.
If not all of the before-mentioned parameters are fulfilled at least one additional positive parameter is needed	
•	Serial lung function testing during antigen exposure periods and days off (exhibiting changes as outlined under SIC as well as restitution of symptoms and impaired lung function during days off) *or*
•	specific inhalative challenge test (exhibiting changes as outlined in Table 2)
•	BAL showing lymphocytosis with ratio of CD4/CD8 < 1 *or*
	typical histopathological findings

Note: we do not recommend the evaluation point system, but rather a careful valuation of the clinical findings and laboratory data in each individual case (see above).

## Case examples

### Case 1 (farmers’ lung)

**Case history**: The 53 year old farmer has suffered for 5 years from cough, progressive shortness of breath during exertion, chills and fever in late evenings and nights during winter months. He always feed his 40 cows hay which was frequently mouldy.

**Physical examination**: Inspiratory crackles on basal lung fields.

Lung function testing: A restrictive ventilatory pattern (i.e. reduced total lung capacity, vital capacity, and lung compliance) and impaired gas exchange parameters.

**IgG antibodies**: High serum concentrations of IgG antibodies for aspergillus species and.

**Specific inhalative challenge test** (Figures. 1D) of this patient suffering from farmers’ lung by a probe of his mouldy hay (Figure 1A); for the outcome see Figure 4.

**Radiological findings**: Patchy opacities on both lower and middle lung fields.

**Bronchoalveolar lavage**: Bronchoalveolar lavage showed leukocytosis (neutrophilia) in the alveoli and small airways in the acute phase followed by an influx of mononuclear cells.

### Case 2 (humidifier lung)

Figure 1C shows humidifier water of a printing plant where several heavily microbially contaminated humidifiers were installed and our 33 year old patient was employed. For chest x-ray findings see Figure 3.

**Case history:** He had complained of flu-like symptoms and chronic productive cough for more than 6 years without seasonal variation and increasing shortness of breath on exertion.

**IgG antibodies:** Serum IgG antibody analysis (Figure 2B) showed extremely high concentrations for the extract of the probe shown in Figure 1C, and lower concentrations for a variety of moulds and bacteria.

**Specific inhalative challenge test**: This was done by means of this humidifier water probe produced after a latency of 4 hours increasing cough, dyspnea, fever, a significant falls of vital capacity and arterial oxygen partial pressure, lasting for 3 hours.

### Prevention and Treatment

The best outcome is offered by early recognition and consistent prevention of further exposures. To avoid the causative agent(s) is also the only effective measure to prevent relapses, the typically progressive disorder and permanently impaired lung function. Corticosteroids may be needed in cases with severe acute courses (starting with 0.5-1 mg prednisone/kg). Less sever acute courses abate without treatment.
